# Folliculin haploinsufficiency causes cellular dysfunction of pleural mesothelial cells

**DOI:** 10.1038/s41598-021-90184-9

**Published:** 2021-05-24

**Authors:** Shouichi Okamoto, Hiroki Ebana, Masatoshi Kurihara, Keiko Mitani, Etsuko Kobayashi, Takuo Hayashi, Yasuhito Sekimoto, Koichi Nishino, Mizuto Otsuji, Toshio Kumasaka, Kazuhisa Takahashi, Kuniaki Seyama

**Affiliations:** 1 Division of Respiratory Medicine, Juntendo University Faculty of Medicine and Graduate School of Medicine, 3-1-3 Hongo, Bunkyo-ku, Tokyo, 113-8431 Japan; 2 Department of Human Pathology, Juntendo University Faculty of Medicine and Graduate School of Medicine, Tokyo, 113-8431 Japan; 3 The Study Group for Pneumothorax and Cystic Lung Diseases, Tokyo, 158-0095 Japan; 4 Department of Thoracic Surgery, Tokyo Metropolitan Bokutoh Hospital, Tokyo, 130-8575 Japan; 5 Pneumothorax Research Center and Department of General Thoracic Surgery, Tamagawa Hospital, Nissan Institute of Medicine, Tokyo, 158-0095 Japan; 6 Department of Pathology, Japanese Red Cross Medical Center, Tokyo, 150-8935 Japan

**Keywords:** Cell adhesion, Mechanisms of disease

## Abstract

Birt–Hogg–Dubé syndrome (BHDS), an autosomal dominant inheritance disease caused by folliculin (*FLCN*) mutations, is associated with lung cysts and spontaneous pneumothorax. The possibility of *FLCN* haploinsufficiency in pleural mesothelial cells (PMCs) contributing to development of pneumothorax has not yet been clarified. Electron microscopy revealed exposed intercellular boundaries between PMCs on visceral pleura and decreased electron density around the adherens junctions in BHDS. To characterize cellular function of PMCs in BHDS patients (BHDS-PMCs), during surgery for pneumothorax, we established the flow cytometry-based methods of isolating high-purity PMCs from pleural lavage fluid. BHDS-PMCs showed impaired cell attachment and a significant decrease in proliferation and migration, but a significant increase in apoptosis compared with PMCs from primary spontaneous pneumothorax (PSP) patients (PSP-PMCs). Microarray analysis using isolated PMCs revealed a significant alteration in the expression of genes belonging to Gene Ontology terms “cell–cell adhesion junction” and “cell adhesion molecule binding”. Gene set enrichment analysis demonstrated that *CDH1*, encoding E-cadherin, was identified in the down-regulated leading edge of a plot in BHDS-PMCs. AMPK and LKB1 activation were significantly impaired in BHDS-PMCs compared with PSP-PMCs. Our findings indicate that *FLCN* haploinsufficiency may affect the E-cadherin-LKB1-AMPK axis and lead to abnormal cellular function in BHDS-PMCs.

## Introduction

Birt–Hogg–Dubé syndrome (BHDS) is an autosomal dominant inheritance disorder characterized by skin fibrofolliculomas, renal tumors, multiple lung cysts, and spontaneous pneumothorax (SP)^1–3^. These clinical features are caused by germline mutations in the folliculin (*FLCN*) gene, which consists of 14 exons, and is located on chromosome 17p11.2^3^. *FLCN* is widely expressed and encodes a protein called folliculin that interacts with various other proteins, including folliculin-interacting proteins 1 and 2 (FNIP1 and FNIP2), p0071 (also called plakophilin-4), and 5′AMP-activated protein kinase (AMPK), a key molecule for energy sensing that negatively regulates mammalian target of rapamycin (mTOR) signaling^4–8^. The *FLCN* gene is considered to function as a tumor suppressor gene, because somatic mutations in the remaining wild-type *FLCN* genes or loss of heterozygosity were identified in BHDS-associated renal tumors^9^, whereas *FLCN* haploinsufficiency appears to be sufficient to initiate uncontrolled cell growth of benign tumors leading to fibrofolliculomas in the skin^10^. Regarding pulmonary manifestations, no neoplastic cells have been detected either on cyst walls or in any other parts of the lungs of BHDS patients. Instead, BHDS lung fibroblasts showed diminished chemotaxis with reduced TGF-β expression in a state of *FLCN* haploinsufficiency^11^. According to these reports^10,11^, other constituent cells of the lungs are also likely to be affected by the *FLCN* haploinsufficiency and consequently, lung cysts may develop and result in the development of pneumothorax.

Pneumothorax develops when air enters the pleural space because of disrupted visceral pleura. The widely accepted mechanism for the occurrence of SP is the rupture of bleb and/or bullae, but logically it also implicates disruption of the mesothelial lining on the most external layer of visceral pleura. The visceral pleural mesothelium is composed of a monolayer of mesothelial cells (MCs) which provide a non-adhesive and protective sealing surface. The gradual accumulation of studies has clarified multifunctional aspects of MCs such as the synthesis of pro-inflammatory cytokines, extracellular matrix proteins and serosal repair, as well as pleural fluid turnover^12,13^. However, neither the role of pleural mesothelial cells (PMCs) in pneumothorax, nor the effect of *FLCN* haploinsufficiency on PMCs in patients with BHDS (BHDS-PMCs) have yet been elucidated. We therefore hypothesized that impaired functions in BHDS-PMCs due to long-lasting *FLCN* haploinsufficiency would contribute to the development of pneumothorax. The purpose of this study was to examine whether *FLCN* haploinsufficiency affects the morphology of PMCs and characterize the cellular functions of PMCs in both BHDS and primary spontaneous pneumothorax (PSP) patients, using PSP-PMCs as a control.

This is the first study to demonstrate that *FLCN* haploinsufficiency affects cellular functions of PMCs, in which impaired E-cadherin-liver kinase B1 (LKB1)-AMPK signaling may have participated. Our findings extend the knowledge of the mechanisms for development of BHDS-associated pneumothorax.

## Materials and methods

### Design

Patients with BHDS (n = 12) or PSP (n = 20) who experienced SP at Juntendo University Hospital, Tokyo Metropolitan Bokutoh Hospital, or Tamagawa Hospital, participated in this study and written informed consent was obtained from all participants. The mean ages were 38.7 (SD = 7.5) for BHDS patients and 25.0 (SD = 5.6) for PSP patients; 33.3% and 70.0% were male in patients with BHDS and PSP, respectively.

The diagnosis of BHDS was established by detection of germline *FLCN* mutations. The *FLCN* mutations of BHDS patients are summarized in Supplementary Table S1 online. The diagnosis of PSP in this study was defined as a patient with pneumothorax meeting the following 2 criteria: (1) age < 40 years and (2) no underlying lung diseases which had been assessed radiologically and pathologically. Electron microscopy was used to examine whether *FLCN* haploinsufficiency affects the morphology of PMCs. To characterize cellular functions of PMCs, we established the methods of isolating high-purity PMCs from patients. This study was approved by the Institutional Review Board of the Juntendo Hospital (No. 18-240) and conducted in accordance with the Declaration of Helsinki. Written informed consent was obtained from all participants.

### Isolation and primary culture of PMCs from pleural lavage fluid

During surgery for SP, a lavage of the pleural space was performed with 100 ml of saline. The pleural lavage fluid was centrifuged at 1500 rpm for 5 min at room temperature. Supernatants were removed and red blood cells were lysed in the lysis buffer (Roche Diagnostics, Mannheim, Germany) for 5 min, followed by centrifugation and washing with phosphate-buffered saline (PBS) twice. The cells were cultured on a collagen-coated 10 cm dish (Corning Incorporated, Corning, NY, USA) with a complete medium, including medium 199 (Sigma-Aldrich, St. Louis, MO, USA) supplemented with 20% fetal bovine serum ([FBS]; Gibco, Grand Island, NY, USA), penicillin (100 U/ml), and streptomycin (100 μg/ml) at 37 °C in a humidified atmosphere of 5% CO_2_. The complete medium was replaced every 3 days.

Cultured cells were detached using 0.05% trypsin/0.2 mM ethylenediaminetetraaceteic acid ([EDTA]; Gibco) and resuspended in PBS with 1% FBS. Cells were incubated for 30 min with mouse anti-human mesothelin IgG antibody, biotinylated by EZ-Link NHS-Biotin (#20217; Thermo Fisher Scientific, Waltham, MA, USA), followed by incubation with Alexa Fluor 488-streptavidin conjugates (#S11223; Thermo Fisher Scientific) for 30 min, and Alexa Fluor 647-conjugated rat anti-human podoplanin antibody for 15 min. Mouse IgG1, κ and rat IgG2a, κ isotype control antibodies were used as isotype-matched control antibodies. Since both mesothelin and podoplanin were positive markers for PMCs, we sorted podoplanin and mesothelin double-positive cells by fluorescence-activated cell sorting (FACS), using a BD FACSAria III cell sorter (BD Biosciences, San Jose, CA, USA). The harvested cells were collected and centrifuged at 1500 rpm for 5 min. After cell density was adjusted, PMCs were cultured for each experiment in the complete medium. All assays were performed without passage because it was difficult to maintain the morphology and proliferation of BHDS-PMCs for more than 2 passages. The list of antibodies used for flow cytometric analysis is shown in Supplementary Table S2 online.

### Electron microscopy

Using a scanning electron microscope (SEM), lung tissues resected during surgery for SP (and cultured PMCs) were fixed with 2% glutaraldehyde solution (TAAB Laboratories Equipment Ltd., Berks, England) in 0.1 M phosphate buffer (pH 7.4), followed by postfixation with 2% OsO_4_ in the same buffer. Fixed specimens were dehydrated with a graded series of ethanol. Dehydrated specimens were transferred into t-butyl alcohol and freeze-dried with an ES-2030 freeze dryer (Hitachi, Tokyo, Japan). After mounting on aluminum stubs with carbon paste, the dried specimens were coated with osmium using an OPC80T osmium plasma coater (Filgen, Inc., Aichi, Japan) and observed with an S-4800 field-emission SEM (Hitachi).

Using a transmission electron microscope (TEM), resected lung tissues were fixed with 2.5% glutaraldehyde, followed by the same postfixation procedure described above. Fixed specimens were dehydrated and embedded in Epok812 (Okenshoji Co., Ltd., Tokyo, Japan). Ultrathin sections were cut and stained with uranyl acetate and lead citrate. These sections were examined with an HT7700 TEM (Hitachi). Reproducibility was confirmed in 3 unrelated samples per group.

### Polymerase chain reaction (PCR)

Isolation of total RNA, complementary DNA synthesis, and PCR were performed with a standard method. The lists of primers used for reverse transcription PCR (RT-PCR) and quantitative real-time reverse transcription PCR (qRT-PCR) analyses are shown in Supplementary Tables S3 and S4 online, respectively. Detailed procedures are described in Supplementary Information online.

### Gene expression microarray analysis

Gene expression microarray analysis was performed in PSP-PMCs (n = 3) and BHDS-PMCs (n = 3) using a SurePrint G3 Human GE v3 8 × 60 K Microarray (Agilent Technologies, Inc., Santa Clara, CA, USA). The array contained 26,083 Enterz gene RNA probes (excluding non-coding RNA). The 6 samples were not well-clustered in each group using the total 26,083 genes and 1 PSP-PMCs sample was clustered into BHDS-PMCs group using 1799 genes when the level of differentially expressed gene was set at a fold change > 1.5 (Supplementary Fig. S1 online). We realized that only this PSP patient remained to be in pneumothorax under the chest tube drainage for about 2 weeks before surgery, i.e., the time when we collected pleural lavage fluid for the isolation of MCs. Therefore, we excluded this sample and used the results from 2 PSP-PMCs and 3 BHDS-PMCs samples. 3378 genes were identified as differential expression genes with a fold change > 1.5; 1637 and 1741 genes were up- and down-regulated, respectively, in BHDS-PMCs compared with PSP-PMCs. We evaluated Gene Ontology (GO) terms significantly affected (*p* < 0.01 and false discovery rate (FDR), *Q* < 0.001) among these genes. The assessment of GO terms and the generation of heatmaps were conducted using GeneSpring14.9.1 (Agilent Technologies, Santa Clara, CA, USA).

### Gene set enrichment analysis

Gene set enrichment analysis ([GSEA], v4.0.3) was performed to assess GO enrichment regarding cell–cell adhesion or cell adhesion by comparing BHDS-PMCs with PSP-PMCs using the entire microarray data set in the GSEA Molecular Signatures Database. GSEA generated each ranked list and the degree of enrichment was indicated by a normalized enrichment score (NES). FDR was also calculated for each NES. FDR, *Q* < 0.025 and NES > 1 was considered significant.

### Flow cytometric analysis of E-cadherin expression

Cultured PMCs were detached with 0.05% trypsin/0.2 mM EDTA and resuspended in PBS with 1% FBS. Cells were incubated for 15 min with allophycocyanin-conjugated mouse anti-human E-cadherin antibody or mouse IgG1, κ isotype control antibody. After incubation, samples were analyzed using a BD LSRFortessa cell analyzer (BD Biosciences), and further analyzed using FlowJo software (Ashland, OR, USA). The mean fluorescence intensity (MFI) ratio was calculated by dividing the MFI of the E-cadherin antibody sample by the MFI of the isotype control sample. An average MFI ratio of 3 samples with E-cadherin antibody was recorded as the representative value.

### Detachment assay

PMCs were seeded onto a collagen-coated 12-well plate (Corning Incorporated) at a density of 1 × 10^5^ cells/well (in 3 replicate wells). Cells with 90% confluence were gently washed with calcium-free PBS twice and incubated with 1 mM ethyleneglycol-*bis*-(β-aminoethyl ether)-*N*,*N*,*N*′,*N*′-tetraacetic acid (EGTA), with a pH of 8.0, for 20 min. Supernatant was collected and the cell number was measured after centrifuge at 1500 rpm for 5 min (see details in Supplementary Information online).

### Cell proliferation assay

A Cell Counting Kit-8 (Dojindo Molecular Laboratories, Inc., Tokyo, Japan) was utilized to evaluate cell proliferation according to the manufacturer’s instructions. Briefly, cells were seeded onto a 96-well plate (Corning Incorporated) in triplicate at a density of 1 × 10^4^ cells/well, and cell proliferation was subsequently evaluated daily for 3 days. To assess cell number, the absorbance at 450 nm was measured in each well using a Model 680 Microplate Reader (Bio-Rad, Hercules, CA, USA), and the mean absorbance of the samples at each incubation time minus the absorbance at time 0 was calculated. The mean absorbance of the 3 replicates in each sample (PSP, n = 4; BHDS, n = 4) was recorded as the representative value.

### Wound cell migration assay

A real-time migration assay was conducted using the IncuCyte ZOOM system (Sartorius, Göttingen, Germany) in 96-well plates according to the manufacturer’s instructions. Briefly, cells were seeded in triplicate at a density of 1 × 10^4^ cells/well and scratch wounds were made using the IncuCyte WoundMaker (Sartorius) after cells reached 90% confluence. The plates were monitored every 3 h via live cell imaging and time-lapse images. Results were presented as wound confluence (%) (i.e., percentage of the wound area occupied by cells). The mean value of the 3 replicates in each sample (PSP, n = 3; BHDS, n = 3) was reported as the representative value.

### Apoptosis assay by flowcytometry

Cell apoptosis was evaluated with flow cytometry using a fluorescein isothiocyanate (FITC)-conjugated Annexin V Apoptosis Detection Kit I (BD Biosciences) according to the manufacturer’s instructions (see details in Supplementary Information online).

### Immunohistochemical staining of cleaved caspase-3

Lung tissues resected during surgery for SP were fixed in 10% buffered formalin and embedded in paraffin. After deparaffination of lung tissue specimens, antigen retrieval was sequentially conducted by autoclave in 10 mM Citrate buffer (pH 6.0) for 15 min at 105 ℃. Samples were incubated with anti-rabbit cleaved caspase-3 antibody (dilution 1:200; #9664, Cell Signaling Technology, Danvers, Massachusetts, USA) for 1 h at room temperature following incubation with Histofine Simple Stain MAX-PO (MULTI) (#424152; Nichirei, Tokyo, Japan) for 30 min at room temperature. Peroxidase staining was visualized with 3,3-diaminobenzidine and hematoxylin was used for counter staining. At least 300 PMCs were counted in each sample and the percentage of PMCs stained with anti-cleaved caspase-3 antibody was calculated in 3 unrelated samples per group.

### Phalloidin immunofluorescence staining and confocal microscopy

Immunofluorescence phalloidin staining was performed as previously described^11^. In our study, PMCs were seeded in triplicate at a density of 3 × 10^5^ cells on a 35 mm glass-bottom dish and cultured in the complete medium until the cells became confluent. Immunofluorescence images were obtained using a Leica TCS SP5 II confocal microscope (Leica Biosystems, Wetzlar, Germany). Reproducibility was verified in 3 unrelated samples in each sample (PSP, n = 3; BHDS, n = 3).

### Measurement of active guanosine triphosphate (GTP)-bound Ras homolog family member A (RhoA) and total RhoA

The levels of active GTP-bound RhoA (RhoA-GTP) and total RhoA were determined using the G-LISA RhoA Activation Assay Biochem Kit (Cytoskeleton Inc., Denver, CO, USA) and the Total RhoA ELISA Assay (Cytoskeleton Inc.), respectively, as done in previous research^11^. In our study, PMCs were seeded at a density of 3 × 10^5^ cells/well onto a collagen-coated 6-well plate (Corning Incorporated) and cultured until approximately 70% confluence was reached. The data were examined in terms of both RhoA-GTP and the RhoA-GTP/total RhoA ratio.

### Western blot analysis of the E-cadherin-LKB1-AMPK signaling pathway

PMCs were seeded at a density of 3 × 10^5^ cells/well on a collagen-coated 6-well plate (Corning Incorporated). After cells reached 90% confluence, the complete medium was changed into serum-free medium, and cells were incubated for 24 h in the absence or presence of 1 mM 5-aminoimidazole-4-carboxamide ribonucleotide ([AICAR]; Wako Pure Chemical Ind. Ltd., Osaka, Japan). Details are provided in Supplementary Information. Supplementary Table S5 online shows the list of antibodies used for Western blot analysis.

### Statistical analysis

Results are expressed as the mean ± SEM unless specified otherwise. Statistical differences between BHDS-PMCs and PSP-PMCs were analyzed using unpaired *t*-tests. A *p* value < 0.05 was considered significant. Statistical analyses were conducted using GraphPad Prsim 7 (GraphPad Software, La Jolla, CA, USA).

## Results

### Ultrastructure of PMCs in vivo

**Figure 1:**
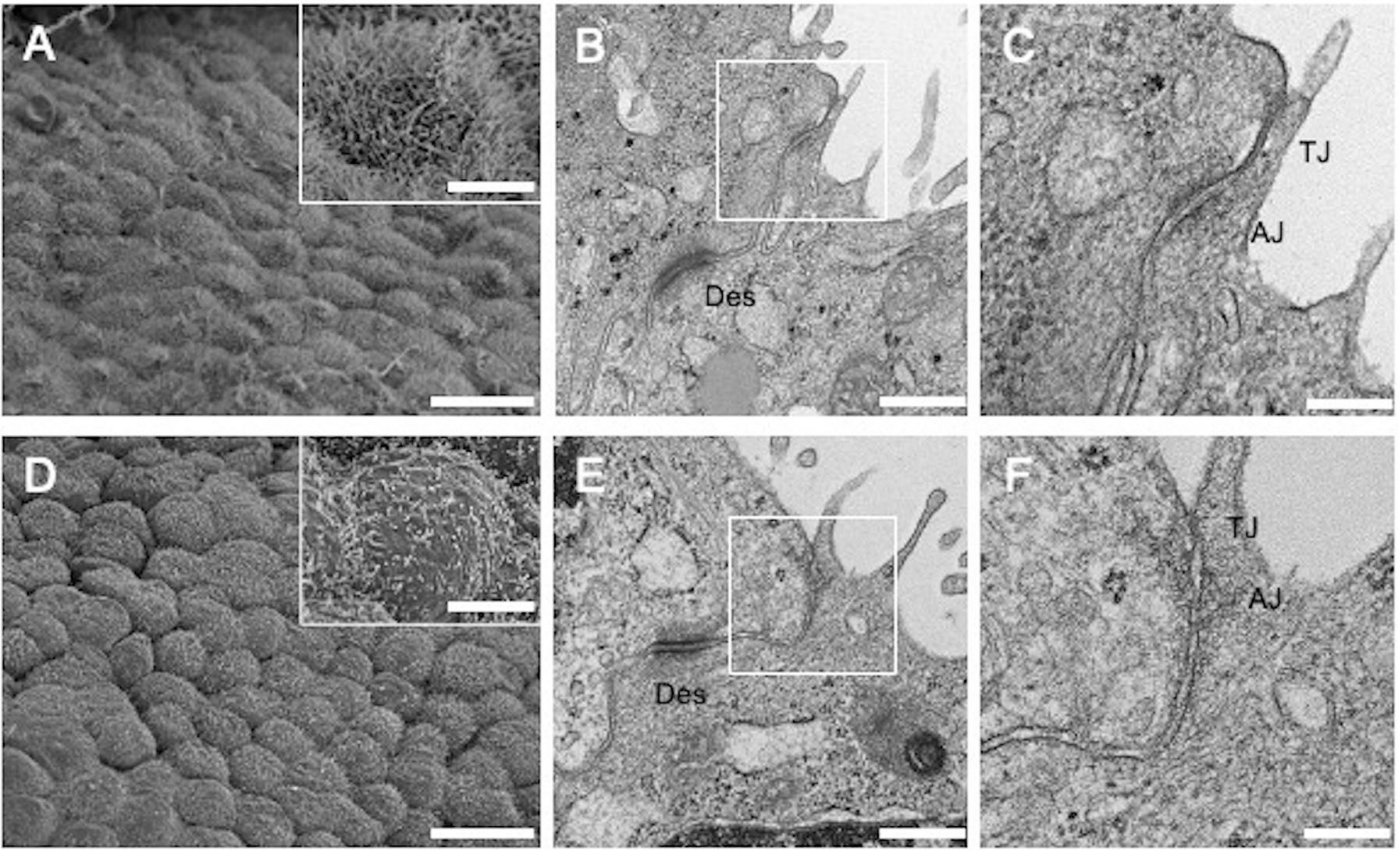
Electron microscopic photographs of visceral pleura from PSP and BHDS patients. (**A**–**C**) Show PSP and Figures (**D**–**F**) show BHDS (n = 3 for both PSP and BHDS). (**A**,**D**) are SEM images. (**B**,**C**,**E**,**F**) Are TEM images of cell–cell junctions. The insets within (**A**,**D**) are magnified views of PMCs. (**C**,**F**) Are the magnified views of the areas indicated by white rectangles within (**B**,**E**), respectively. Scale bars represent 20 μm (**A**,**D**), 5 μm (the insets within **A**,**D**), 500 nm (**B**,**E**) and 200 nm (**C**,**F**). *AJ* adherens junction, *Des* desmosome, *SEM* scanning electron microscope, *TEM* transmission electron microscope, *TJ* tight junction.

To elucidate the possible morphological differences between PSP- and BHDS-PMCs, we initially examined visceral pleura in PSP and BHDS patients using SEM and TEM. The SEM revealed that the pleural surface in PSP patients had tightly laid PMCs with numerous microvilli (Fig. 1A). It had the appearance of cobblestones with a slightly uneven surface. In contrast, the pleural surface in BHDS patients appeared to be more cuboidal with exposed intercellular boundaries, and each PMC had a smaller number of microvilli that were shorter in length than those of PSP-PMCs (Fig. 1D). The TEM images of cell–cell junctions in BHDS patients (Fig. 1E,F) revealed decreased electron density around the adherens junctions corresponding to the attachment of actin filaments compared with those of PSP-PMCs (Fig. 1B,C), although neither tight junctions nor desmosomes showed apparent differences. These findings suggest that there are some morphological abnormalities associated with the cell adherens junctions in BHDS-PMCs.

### Isolation of PMCs from pleural lavage fluid

**Figure 2:**
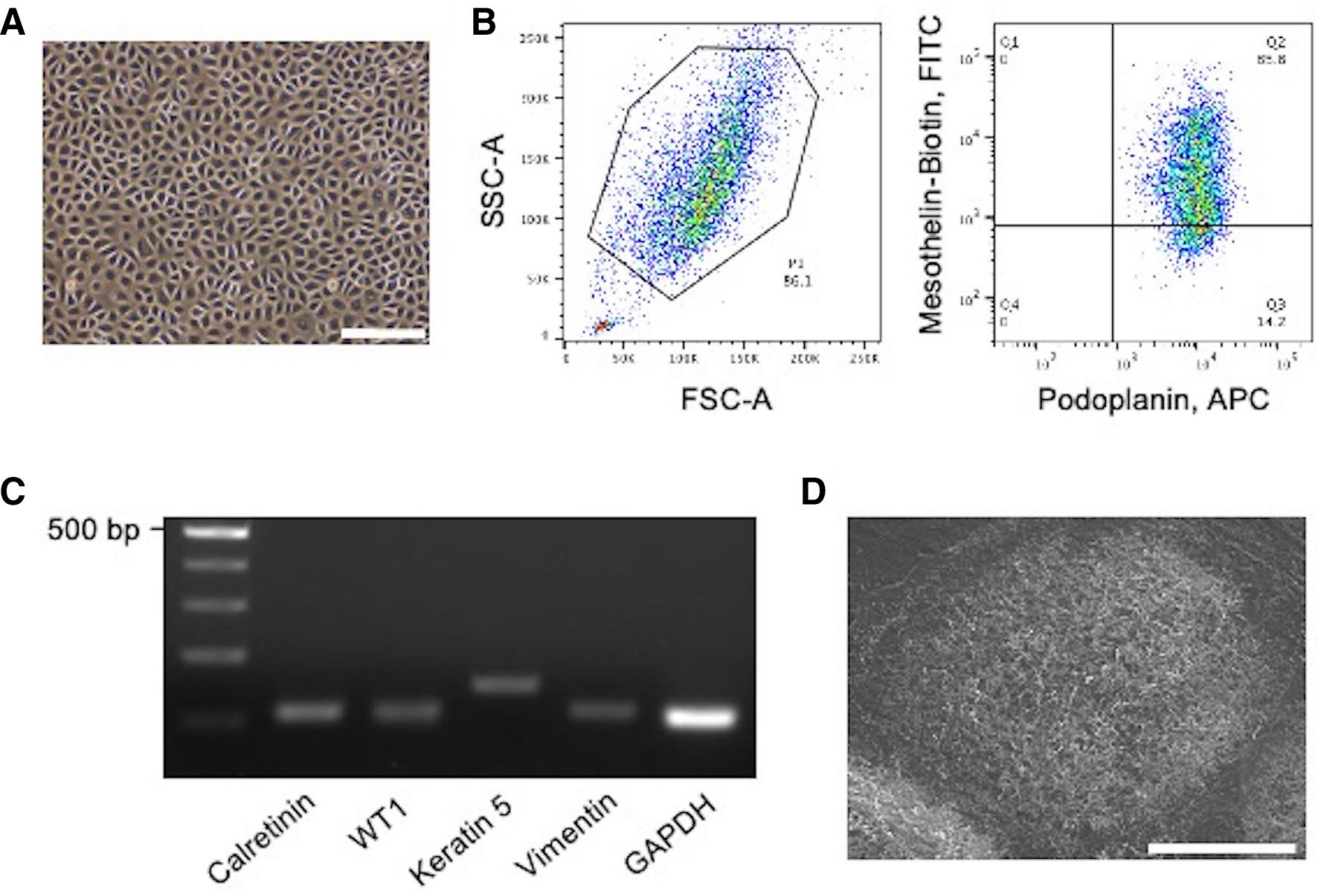
Isolation and characterization of human PMCs from pleural lavage. (**A**) Representative photomicrograph of confluently growing cells obtained from pleural lavage in PSP patient. (**B**) Representative fluorescence-activated cell sorting dot plots of cultured cells. The left panel shows plots of the forward-scattered (horizontal) versus side-scattered (vertical) axes. The right panel represents the fluorescent intensities of APC (conjugated with the anti-podoplanin antibody) and FITC (conjugated with the biotinylated anti-mesothelin antibody), respectively. The Q2 area indicates the gateway for sorting PMCs (podoplanin-positive and mesothelin-positive cells). (**C**) The expression of MC markers (calretinin, *WT1*, keratin 5, and vimentin) examined in PSP-PMCs by RT-PCR. *GAPDH* was amplified as a control gene for RT-PCR. Gel electrophoresis of amplified products showed a discrete band with expected fragment size from each reaction. (**D**) SEM image of PSP-PMCs showing numerous microvilli characteristic of MCs. Scale bars represent 200 μm (**A**) and 20 μm (**D**), respectively. *APC* allophycocyanin, *bp* base pair, *FITC* fluorescein isothiocyanate, *FSC-A* forward scatter area, *GAPDH* glyceraldehyde 3-phosphate dehydrogenase, *SSC-A* side scatter area, *WT1* Wilms’ tumor 1.

To examine the cellular characteristics of PMCs, they were isolated from pleural lavage fluid during surgeries for pneumothorax (PSP, n = 20; BHDS, n = 12). The primary cell culture yields from 100 ml of pleural lavage fluid on a 10 cm dish were 10.4 ± 7.0 × 10^6^ from PSP patients and 8.3 ± 3.6 × 10^6^ from BHDS patients (mean ± SD). Cultured cells showed a cobblestone appearance suggesting the morphology of mesothelial cells (MCs) (Fig. 2A). Flow cytometry revealed that most cultured cells were both podoplanin- and mesothelin-positive cells (Fig. 2B). We sorted the double-positive cells by flow cytometry and then examined if they had cellular characteristics of MCs. RT-PCR demonstrated the expression of various MC markers such as calretinin, Wilms’ tumor 1 (*WT1*), keratin 5, and vimentin (Fig. 2C). SEM demonstrated a cuboidal cell shape with numerous microvilli, another characteristic of MCs (Fig. 2D). Based on these findings, we concluded that our sorted cells were PMCs and could be utilized to examine the effects of *FLCN* haploinsufficiency on BHDS-PMCs. The final yields of PMCs from BHDS and PSP patients respectively were 2.2 ± 1.2 × 10^6^ and 2.5 ± 1.1 × 10^6^ (mean ± SD), with recovery rates of 25.9% ± 6.4 and 26.9% ± 9.1 (mean ± SD) of the primary cultured cells on a 10 cm dish, respectively.

### Gene expression analyses revealed alterations in cell–cell adhesion junction in BHDS-PMCs

**Figure 3:**
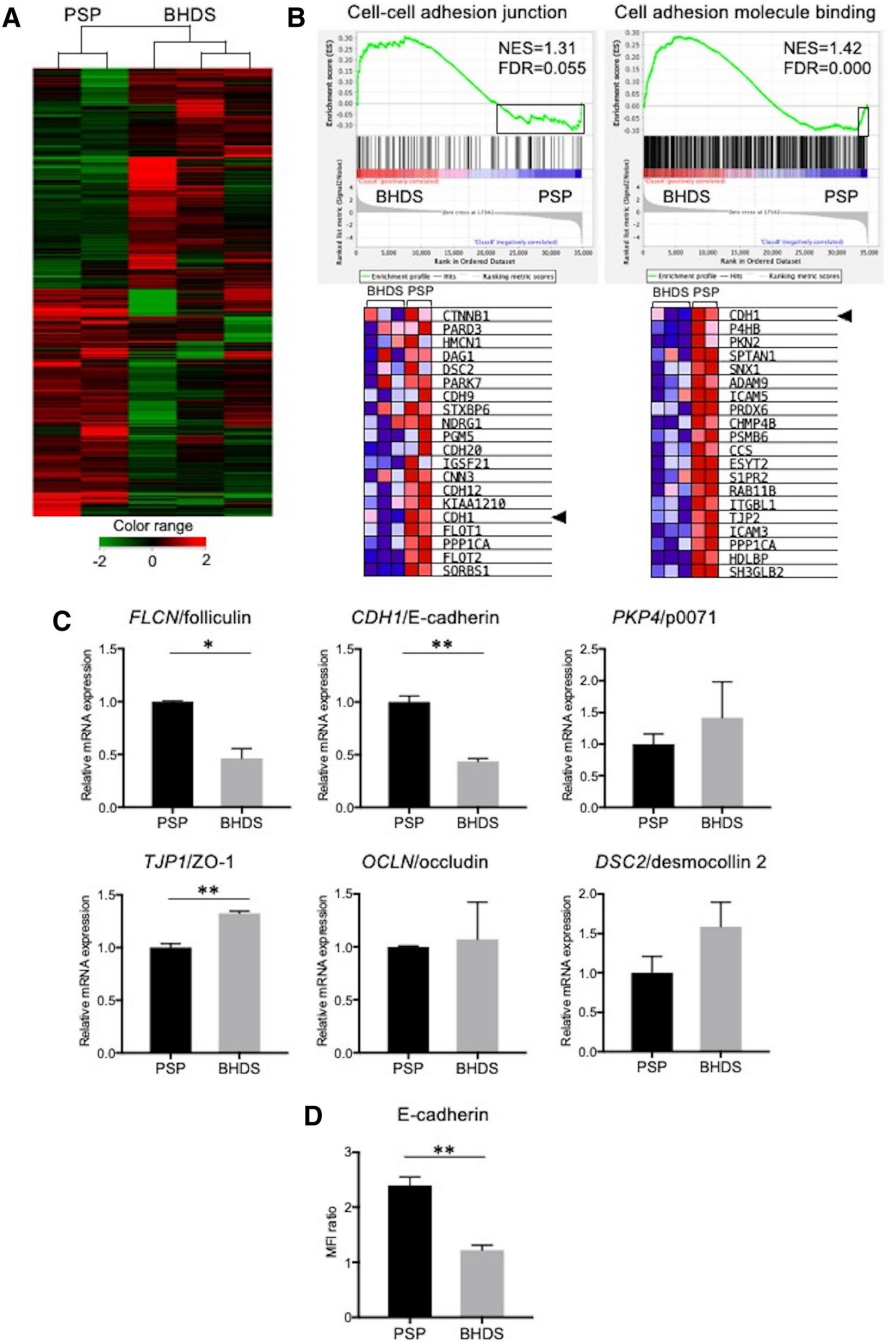
Gene expression microarray analysis and the validation of microarray data by qRT-PCR. (**A**) Hierarchical clustering analysis in PSP-PMCs (n = 2) and BHDS-PMCs samples (n = 3). A dendrogram, consisting of 3378 genes with a fold change > 1.5, shows the grouping of genes based on the similarity between them. Increased and decreased gene expression is shown from red to green, respectively. The color-range bar indicates a log2 fold change. The analysis illustrated that PSP-PMCs and BHDS-PMCs separated into 2 different groups. The data were processed and analyzed using GeneSpring14.9.1 (http://genespring-support.com/). (**B**) Results of GSEA of BHDS-PMCs compared to PSP-PMCs utilizing the GO terms “cell–cell adhesion junction” and “cell adhesion molecule binding” (upper panel). The bar-code plots indicate the position of each gene within the expression data, rank-sorted by its association with BHDS-PMCs, with the red and blue colors representing overexpression and underexpression in the mRNA, respectively. The lower panel displays heat maps of the bottom 20 genes in BHDS-PMCs, which are indicated by black rectangle within the upper panel. *CDH1*, which encodes E-cadherin (the main constituent of adherens junction), was the 5th and 20th lowest downregulated gene in the 2 gene sets, respectively (arrowheads). (**C**) Findings from testing of the mRNA expression levels of *FLCN* and various other genes as measured for cell–cell junction by qRT-PCR. The vertical axes indicate the relative ratios of mRNA expression (BHDS/PSP) normalized against *GAPDH*. All reactions were conducted in triplicate for each sample (PSP, n = 2; BHDS, n = 3). (**D**) The expression level of E-cadherin by flow cytometry was tested for group differences (PSP, n = 3; BHDS, n = 3). Data are shown as mean ± SEM. * = *p* < 0.05; ** = *p* < 0.01 (unpaired *t-*test). *FDR* false discovery rate, *GAPDH* glyceraldehyde 3-phosphate dehydrogenase, *GSEA* gene expression microarray analysis, *MFI* mean fluorescence intensity, *NES* normalized enrichment score, *ZO-1* zonula occludens-1.

To assess the possible changes in gene expression associated with the inborn status of *FLCN* haploinsufficiency, we performed gene expression microarray analysis using RNA of PSP- and BHDS-PMCs. Based on the expression profile, PSP-PMCs (n = 2) and BHDS-PMCs (n = 3) samples were separated into 2 groups (Fig. 3A). The 1637 and 1741 genes (from a total of 3378 genes examined) were > 1.5-fold up- and down-regulated, respectively, in BHDS-PMCs compared to PSP-PMCs. A Gene Ontology (GO) analysis of gene expression demonstrated that the GO term element “positive regulation of cell adhesion” (GO: 0045785) was listed as one of the statistically significant terms (*p* < 0.01 and false discovery rate (FDR), *Q* < 0.001), as shown in Supplementary Table S6 online. From the electron microscopic results and GO analysis, we performed GSEA focused on cell–cell adhesion or cell adhesion using GO terms. The GSEA demonstrated that more than half of the genes included in the GO term elements, “cell–cell adherens junction” and “cell adhesion molecule binding” were upregulated in BHDS-PMCs (FDR, *Q* = 0.055, 0.000, respectively) (Fig. 3B, upper row). However, we noted that the expression of *CDH1* (which encodes E-cadherin), the main constituent of adherens junctions^14^, was identified in the downregulated leading edge of the GSEA plot in BHDS-PMCs (Fig. 3B, bottom row). Since previous research has reported that the inducible *FLCN* knockdown in primary mouse alveolar epithelial cells caused decreased E-cadherin expression resulting in their apoptosis^15^, we decided to further examine the consequences of lowered E-cadherin expression in BHDS-PMCs.

We validated the results of the microarray analysis regarding the genes which encode major constituents of intercellular junctions. qRT-PCR confirmed the significantly decreased messenger RNA (mRNA) expressions of folliculin and E-cadherin in BHDS-PMCs (Fig. 3C). Conversely, the mRNA expression of zonula occludens-1 (a constituent of tight junctions) slightly, but significantly increased in BHDS-PMCs, whereas mRNA expression of other components of cellular junctions including p0071 (a constituent of adherens junctions), occludin (a component of tight junctions), and desmocollin 2 (a constituent of desmosomes) showed no significant changes (Fig. 3C). Furthermore, flow cytometry demonstrated a significant decreased mean fluorescence intensity of E-cadherin in BHDS-PMCs compared with PSP-PMCs (Fig. 3D), confirming the decreased cell surface expression of E-cadherin in BHDS-PMCs.

### Impaired cell attachment in BHDS-PMCs

**Figure 4:**
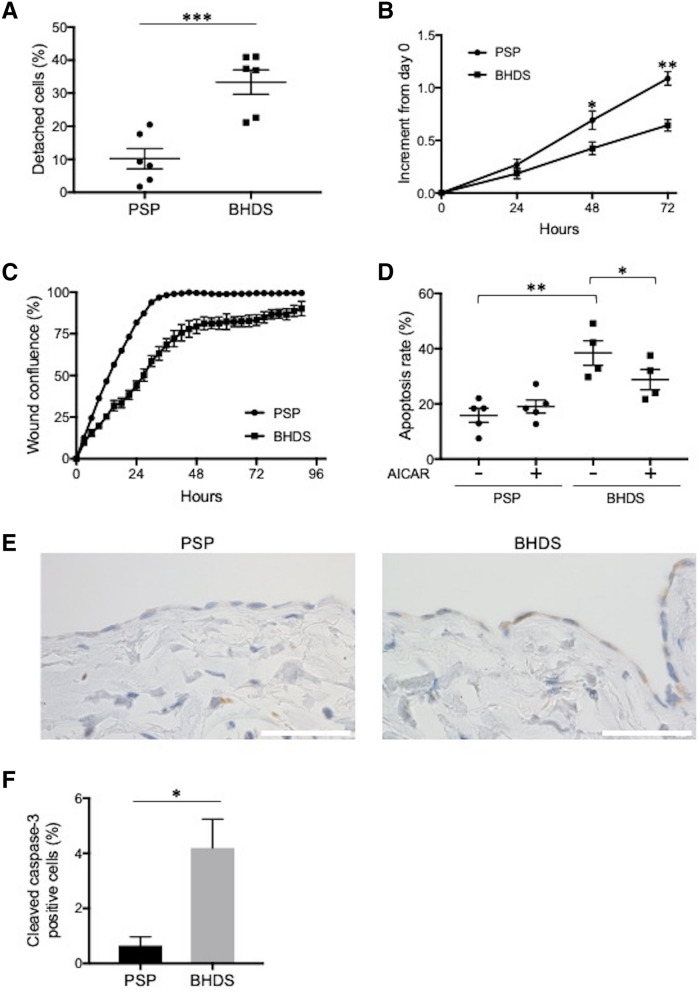
Comparison of cellular characteristics between PSP- and BHDS-PMCs. (**A**) Plot of cell detachment assay findings. PMCs growing nearly confluent on 12-well culture dish were incubated with 1 mM EGTA for 20 min. The percentage of detached cells was computed by dividing the number of detached PMCs by the total number of PMCs. The vertical axis indicates the percentage of detached cells. The circles and squares within the plot depict the mean values of the 3 replicates in each sample (PSP, n = 6; BHDS, n = 6). (**B**) Plot of proliferation assay results. The vertical axis indicates the increase of absorbance at 450 nm from baseline (increment from day 0). 1 × 10^4^ PMCs at passage 1 were seeded on 96-well culture with triplicate (PSP, n = 4; BHDS, n = 4). (**C**) Plot of wound cell migration assay findings. 1 × 10^4^ PMCs at passage 1 were seeded on 96-well culture with triplicate. The vertical axis indicates the percentage of wound confluence (PSP, n = 3, BHDS, n = 3). (**D**) Plot of results from apoptosis assay by flow cytometry. PMCs growing nearly confluent on 12-well culture dish were incubated in complete medium with or without 0.5 mM AICAR for 24 h, then harvested by 0.05% trypsin/0.2 mM EDTA and the percentage of apoptotic cells was determined. The vertical axis indicates the percentage of apoptotic cells. The circles and squares within the plot depict the mean values of the 3 replicates in each sample (PSP, n = 5; BHDS, n = 4). (**E**) Representative photomicrographs showing the immunostaining of cleaved caspase-3 in paraffin-embedded lung tissues of PSP and BHDS patients. PMCs are lining on the surface of visceral pleura. In BHDS (a right photomicrograph), area where several cleaved caspase-3-positive PMCs is gathering on the visceral pleura. Scale bars represent 50 μm. (**F**) The ratio of cleaved caspase-3-positive PMCs in the resected lung tissues. At least 300 PMCs on visceral pleura were evaluated in each lung tissue (PSP, n = 3; BHDS, n = 3). All data are shown as mean ± SEM. The central horizontal lines in Figures (**A**,**D**) indicate the position of the mean values. * = *p* < 0.05; ** = *p* < 0.01; *** = *p* < 0.001 (unpaired *t-*test). *AICAR* 5-aminoimidazole-4-carboxamide ribonucleotide, *EDTA* ethylenediaminetetraacetic acid, *EGTA* ethyleneglycol-*bis*-(β-aminoethyl ether)-*N*,*N*,*N′*,*N′*-tetraacetic acid.

E-cadherin is one of the calcium-dependent cell adhesion molecules and a key component of adherens junctions^14,16^. The binding of E-cadherin is inhibited by calcium-chelating agents such as EDTA and EGTA, which induce the opening of trans-interactions of E-cadherin in adjacent cells. EGTA binds Ca^2+^ with a greater affinity than other divalent cations, and detaches cells in proper concentration^17^. We hypothesized that downregulation of E-cadherin expression in BHDS-PMCs could lead cells to detach from the surface of culture dishes in even lower EGTA concentrations than is typical. To verify our hypothesis, we incubated both types of PMCs with 1 mM EGTA (pH 8.0) based on our preliminary experiment and the report by Cox et al.^17^. The percentage of detached BHDS-PMCs was significantly higher than those of PSP-PMCs (Fig. 4A). There was no significant difference in the number of dead cells using trypan blue staining (data not shown). The significant result suggests that BHDS-PMCs have impaired cell attachment, which might be due to the downregulation of E-cadherin expression in the *FLCN* haploinsufficiency state.

### Proliferation, migration, and apoptosis in BHDS-PMCs

Previous studies have reported that inducible *FLCN* deletion in mouse alveolar epithelial cells resulted in increased apoptosis attributable to the impaired function of the E-cadherin-LKB1-AMPK axis^15^. Therefore, we evaluated whether basic cellular functions in BHDS-PMCs are altered because of *FLCN* haploinsufficiency.

A proliferation assay showed a significant decrease in cell growth of BHDS-PMCs (Fig. 4B). A wound cell migration assay demonstrated a significant delay of wound healing in BHDS-PMCs over 90 h (Fig. 4C). Furthermore, flow cytometric analysis showed a significant increase of apoptosis in BHDS-PMCs (Fig. 4D). AICAR, an activator of AMPK, significantly reduced the apoptotic rate in BHDS-PMCs, but did not in PSP-PMCs (Fig. 4D). AICAR did not ameliorate the proliferation in either PSP-PMCs or BHDS-PMCs (data not shown). Immunohistochemical analysis of resected lung tissues demonstrated that the expression level of cleaved caspase-3, a marker for cells undergoing apoptosis in the caspase-dependent pathway, increased in PMCs on the visceral pleura from BHDS lung tissues compared with those from PSP lung tissues (Fig. 4E). The percentage of cleaved caspase-3-positive PMCs on the visceral pleura significantly increased in BHD lung tissues compared with PSP lung tissues (Fig. 4F). These results suggest that the long-lasting *FLCN* haploinsufficiency state in BHDS patients causes dysfunction of basic PMC cellular properties.

### Aberrant F-actin formation and diminished RhoA activity in BHDS-PMCs

**Figure 5:**
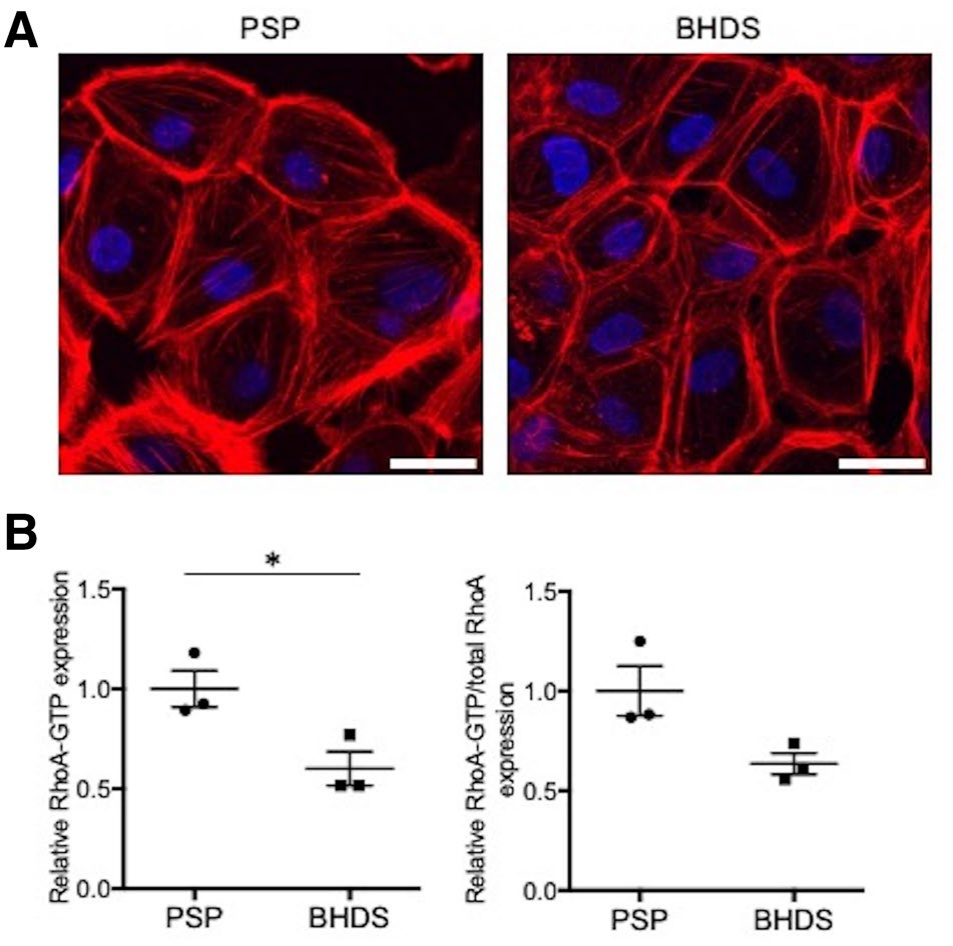
Comparison of F-actin formation and RhoA activity between PSP- and BHDS-PMCs. (**A**) Representative confocal photomicrographs of phalloidin staining in PSP- and BHDS-PMCs (PSP, n = 3; BHDS, n = 3). At passage 1, 3 × 10^5^ PMCs were seeded onto a 35 mm glass-bottom dish until the cells became confluent. Scale bars represent 20 μm. (**B**) Plot of the levels of active RhoA-GTP and total RhoA. The vertical axis in the left plot indicates the relative RhoA-GTP expression when normalized by the mean level of RhoA-GTP in PSP-PMCs. At passage 1, 3 × 10^5^ PMCs were seeded on a collagen-coated 6-well plate, cultured until approximately 70% confluence, and then utilized for the measurement. The vertical axis in the left panel indicates the relative ratio of RhoA-GTP when normalized by the mean level of RhoA-GTP in PSP-PMCs. The vertical axis in the right plot indicates the relative ratio of RhoA-GTP/total RhoA when normalized by the mean level of RhoA-GTP/total RhoA in PSP-PMCs. The circles and squares indicate the measured values of each sample (PSP, n = 3; BHDS, n = 3) and the central horizontal lines indicate the position of mean values. The data are shown as mean ± SEM. * = *p* < 0.05 (unpaired *t-*test).

RhoA is a ubiquitously expressed small GTPase suggested to be essential for stress fiber formation, epithelial cell–cell contacts, and cytokinesis. Previous studies have shown that loss of the *FLCN* gene led to the dysregulation of RhoA signaling. RhoA-GTP, an active form of RhoA, was increased by about 30% in kidney cancer cells with knockdown of *FLCN*, which co-localizes and interacts at the midbody during cytokinesi*s* with p0071^7^. Conversely, RhoA-GTP was decreased by 60% in a *FLCN*-null kidney cancer cell line (UOK257) compared with a *FLCN*-expressing isogenic kidney cancer cell line (UOK257-2)^8^. Furthermore, *FLCN* haploinsufficiency has been found to cause the decrease of the organization of actin stress fibers as well as RhoA-GTP in BHDS lung fibroblasts^11^. Accordingly, we evaluated the organization of actin filaments and RhoA activity in BHDS-PMCs. Phalloidin staining seemingly showed the decreased organization of actin stress fibers, both in the cytoplasm and around the periphery of cells in BHDS-PMCs compared with PSP-PMCs (Fig. 5A). Additionally, the level of RhoA-GTP was significantly diminished in BHDS-PMCs (*p* = 0.032) and the ratio of RhoA-GTP/total RhoA showed a near significant decrease in BHDS-PMCs (*p* = 0.055) compared with PSP-PMCs (Fig. 5B). These results indicate that the *FLCN* haploinsufficiency state caused aberrant F-actin formation and diminished RhoA activity in PMCs as we previously reported in lung fibroblasts^11^.

### *FLCN* haploinsufficiency impaired AMPK and LKB1 phosphorylation in PMCs

*FLCN* forms a complex with FNIP1, FNIP2 and AMPK, and both *FLCN* and FNIP1 could function as substrates for AMPK^4^. *FLCN* deletion in mouse alveolar epithelial cells, in the mammary glands cell line (NMuMG) significantly decreased AMPK phosphorylation^15^. AMPK phosphorylation is required to regulate cell survival and energy metabolism, to strengthen the epithelial barrier and promote the assembly of tight junctions and adherens junctions^18–20^. Conversely, force applied to E-cadherin activates AMPK through LKB1 recruitment to the cadherin adhesion complex^21^. AMPK is activated with phosphorylation of threonine 172 (also known as Thr172) by LKB1 in response to energy stress, such as nutrient starvation.

**Figure 6:**
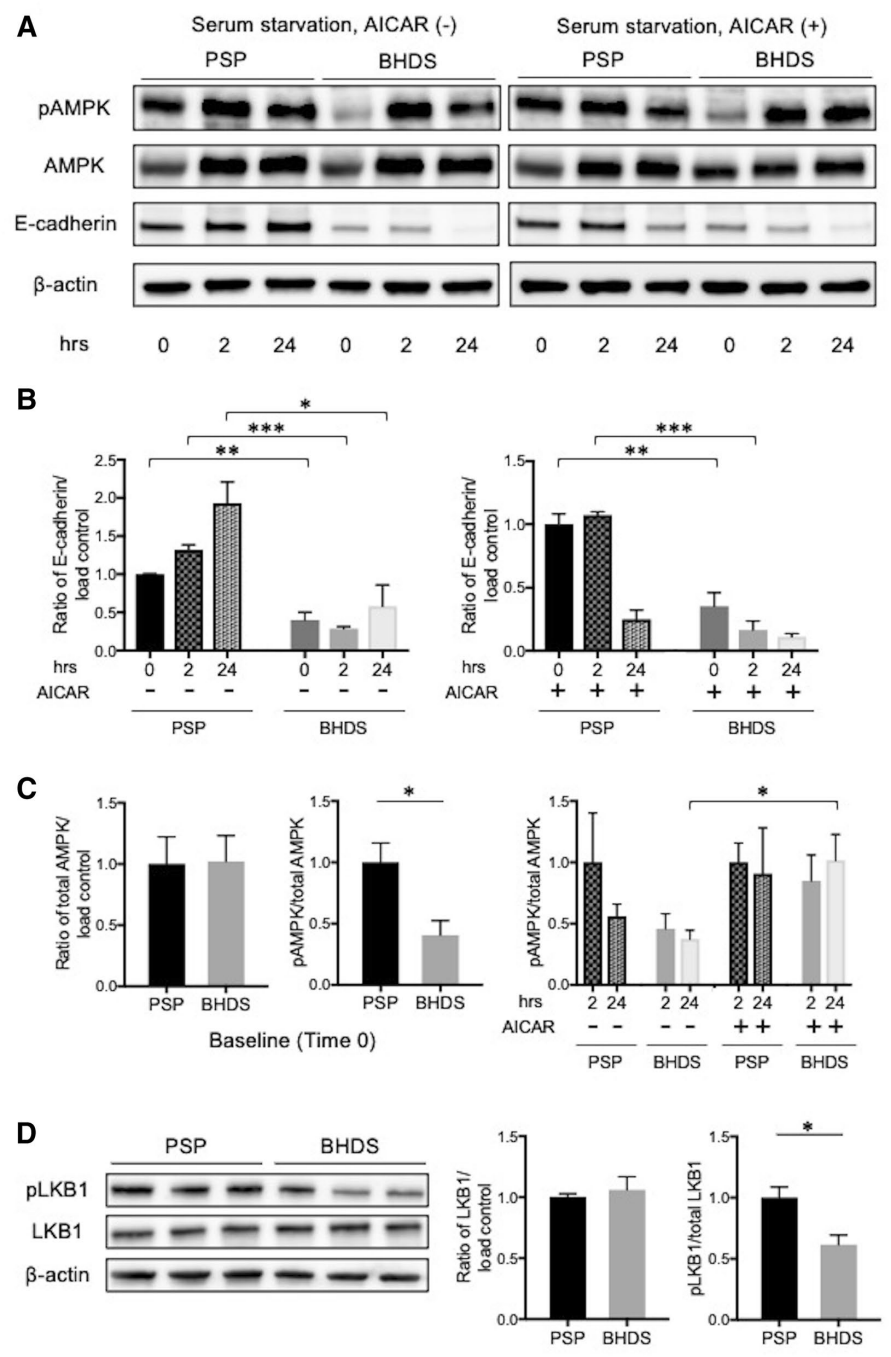
Comparison of status of the E-cadherin-LKB1-AMPK axis between PSP- and BHDS-PMCs. (**A**) Representative findings from Western blot analysis for phospho-AMPK (pAMPK), total AMPK, E-cadherin, and β-actin (load control)(PSP, n = 3; BHDS, n = 3). PMCs were serum-starved for 24 h without or with 1 mM AICAR and then harvested for analysis. (**B**) Graphs of results from quantitative analysis of E-cadherin expression on Western blot without (left panel) or with 1 mM AICAR (right panel). The vertical axes represent the ratio of E-cadherin/β-actin when normalized by the mean value of E-cadherin/β-actin at baseline in PSP-PMCs. Data are shown as mean ± SEM from 3 independent experiments. (**C**) Graphs of results from quantitative analyses of total AMPK and pAMPK/total AMPK ratios on Western blot. The ratios of total AMPK/β-actin (left panel) and pAMPK/total AMPK at baseline (middle panel) in PSP- and BHDS-PMCs are shown. The right panel shows the ratio of pAMPK/total AMPK without and with 1 mM AICAR. Each vertical axis represents the ratio when normalized by the mean value of pAMPK/total AMPK in PSP-PMCs at 2 h after incubation. Data are shown as mean ± SEM. (**D**) Representative findings from Western blot analyses for phospho-LKB1 (pLKB1), total LKB1, and β-actin (left panel), quantitative analyses of total LKB1 (middle panel), and the ratio of pLKB1/total LKB1 in the complete medium (right panel). All data are shown as mean ± SEM. * = *p* < 0.05; ** = *p* < 0.01; *** = *p* < 0.001 (unpaired *t-*test). *AICAR* 5-aminoimidazole-4-carboxamide ribonucleotide.

We therefore examined the E-cadherin-LKB1-AMPK signaling pathway in PMCs by Western blot analysis. The expression level of E-cadherin in BHDS-PMCs at baseline (Time 0) decreased significantly (Fig. 6A,B), in accordance with results of the qRT-PCR and flow cytometric analyses (Fig. 3C,D). The E-cadherin expression increased in PSP-PMCs at 2 and 24 h after serum starvation but did not in BHDS-PMCs (Fig. 6A,B, left). When PMCs were stimulated with 1 mM AICAR, no increase of E-cadherin expression was found in BHDS-PMCs whereas the E-cadherin expression in PSP-PMCs significantly decreased at 24 h (Fig. 6A,B, right), suggesting that AICAR might be harmful to *FLCN*-preserved PSP-PMCs.

Next, we investigated AMPK phosphorylation in BHDS-PMCs with a *FLCN* haploinsufficiency state. Although there was no significant difference in the expression level of total AMPK between PSP- and BHDS-PMCs, AMPK phosphorylation at baseline (Time 0) was significantly diminished in BHDS-PMCs compared with that in PSP-PMCs (Fig. 6A,C, left and middle). AICAR significantly enhanced AMPK phosphorylation in BHDS-PMCs at both 2 and 24 h after serum starvation, whereas no increased phosphorylation of AMPK was found in PSP-PMCs (Fig. 6A,C, right).

LKB1 is an upstream kinase in an AMPK signaling cascade^22^ and co-localized with E-cadherin at adherens junctions^21,23^. Although previous research has reported that the expression of LKB1 in mouse alveolar epithelial cells decreased with the lack of *FLCN* expression^15^, no significant difference in LKB1 expression was found between PSP- and BHDS-PMCs (Fig. 6D, left and middle). LKB1 is phosphorylated by protein kinases at various sites^24–26^, but protein kinase C-ζ phosphorylation of LKB1 at serine 428 (Ser428), lead to metformin-enhanced AMPK activation^24^. When we assessed the phosphorylation at Ser428 on LKB1, we found that baseline LKB1 phosphorylation at Ser428 was significantly diminished in BHDS-PMCs compared with that in PSP-PMCs (Fig. 6D, left and right). These results suggest that *FLCN* haploinsufficiency might hamper the E-cadherin-LKB1-AMPK signaling pathway in BHDS-PMCs. The decreased expression of E-cadherin could impair the phosphorylation of co-localizing LKB1, which consequently results in diminished AMPK phosphorylation.

## Discussion

Our study is the first to reveal that *FLCN* haploinsufficiency causes alterations in the morphology of PMCs, and impairs their cellular functions, including proliferation, migration, attachment, and organization of cytoskeleton. The E-cadherin-LKB1-AMPK signaling pathway may be involved in those functions. We first established the FACS-based methods of isolating PMCs with high purity from pleural lavage fluid. We discovered an opportunity during a pneumothorax surgery in which pleural lavage was necessary. Furthermore, we confirmed that isolated PMCs expressed various MC markers and showed characteristic appearances of MCs, by both inverted microscope and SEM.

Previous studies of isolation and characterization of human PMCs have been limited. Zolak et al. reported obtaining PMCs from the explanted lungs of patients with idiopathic pulmonary fibrosis by surface scraping of the visceral pleura^27^. Kienzle et al. analyzed pleural effusion fluid obtained from patients with chest drains after lung resection or transplantation surgeries, and flow cytometry directly identified free-floating PMCs in the fluid (by size analysis) as well as negativity for CD45 (also known as leucocyte common antigen)^28^. *WT1*-positive and CD71 (transferrin receptor)-positive cells were regarded as activated PMCs in their study, but were not utilized for further investigation. In several other studies, plural fluids collected during pneumothorax surgeries were seeded after centrifuge, and cultured PMCs were examined for characteristics of MCs in terms of appearance in culture, type of intermediate filaments, or reactions with antibodies such as cytokeratin, vimentin, and E-cadherin^29–31^. However, those PMCs were utilized in subsequent experiments for 2 to 5 passages (mean of 3 passages) without purification, mainly due to declined cell growth and cellular senescence^29–31^.

Our FACS-based method established in this study showed similar yields of PMCs, with higher purity than those used in previous studies^28,32^. Additionally, we were able to utilize PSP-PMCs in subsequent experiments for at least 3 to 5 passages. In contrast, BHDS-PMCs were unable to maintain their morphology and viability for more than 2 passages. Although the exact mechanisms remain unsolved, that may be a consequence of a long-standing status of *FLCN* haploinsufficiency.

The mechanisms for cystogenesis in the lungs of BHDS patients have been investigated, but those for BHDS patients developing pneumothorax have not yet been clarified. The occurrence of pneumothorax essentially results from disruption of the mesothelium. Ohata et al. investigated the surface of emphysematous bullae in patients with PSP using SEM^33^. They found that the development of pneumothorax did not necessarily require the rupture of bullae, and the sloughing of PMCs’ lining on the most external layer of pleura inevitably resulted in pneumothorax.

In this context, our results regarding the alterations in morphology of PMCs suggest that the fragility of cell–cell junctions together with impaired cellular functions appear to have substantial significance in the development of pneumothorax in BHDS. We demonstrated that BHDS-PMCs have decreased expression of E-cadherin and hence were easy to detach from culture dishes. Loss of cell adhesion in epithelial cells provokes detachment-induced apoptosis, which is called anoikis^34^, and it can be inhibited by maintaining E-cadherin-mediated cell–cell adhesion in spite of loss of cell–matrix interactions in human breast cancer cell lines^35^. We found that BHDS-PMCs showed a higher rate of apoptosis than PSP-PMCs when detached from culture dishes, which was significantly inhibited by the addition of AICAR. Furthermore, we demonstrated a higher apoptotic rate in PMCs on the visceral pleura from BHDS lung tissues. Based on these results, we speculate that both impaired cell attachment and the increased rate of apoptosis in BHDS-PMCs in vitro and in vivo have a significant role in the development of pneumothorax in BHDS patients.

Slower proliferation and migration in BHDS-PMCs are further expected to have an additional role in the development of pneumothorax in BHDS patients. It is widely recognized that the main role of the mesothelium is to facilitate intracoelomic motions, and function as a protective barrier against physical damage and microorganisms. However, recent studies have revealed that the mesothelium is also involved in fluid transport, tissue repair, the regulation of inflammation, antigen presentation, and the prevention of tumor cell adhesion^36^. Pleural lavages of patients with PSP showed substantial inflammatory reactions because the concentrations of cytokines IL-5, IL-6, IL-8, IL-12p40, and TNF-α were significantly increased in comparison with those of patients with essential hyperhidrosis (control group)^37^.

Peritoneal MCs, when activated by the macrophages which exist in intracoelomic fluids, secrete various inflammatory mediators^38–40^. These data indicate that pneumothorax evokes inflammatory reactions through the activation of PMCs. Peritoneal MCs also secrete extracellular matrix molecules and various growth factors which induce cell proliferation and migration in response to injury^36,41^. Injury to the mesothelium causes migration of peritoneal MCs from the wound edge to the center of the wound as well as incorporation of free-floating MCs in the peritoneal fluid into the regenerating mesothelium^42^. Furthermore, PMCs have both procoagulant and fibrinolytic activity implicated in tissue repair by producing cellular initiators such as tissue factor, tissue plasminogen activator, urokinase plasminogen activator, and plasminogen activator inhibitors-1 and -2^29,30,43,44^. PMCs are required to exert these activities seamlessly in a well-coordinated fashion once the mesothelium is damaged. If PMCs fail to work well, disruption of the mesothelium will develop, which inevitably results in pneumothorax. In future experiments, we need to address how multiple aspects of PMCs function aside from proliferation and migration, especially how functions related to tissue repair are altered by FLCN haploinsufficiency.

Our results showed that *FLCN* is necessary for PMCs to maintain their fundamental cellular functions, presumably through the E-cadherin-LKB1-AMPK signaling pathway. *FLCN* as a tumor suppressor gene is a conserved negative regulator of AMPK, but the functional relationship between *FLCN* and AMPK is equivocal based on previous investigations on non-tumor cells using in vitro and in vivo systems. Loss of *FLCN* induced decreased AMPK phosphorylation in NMuMG cells and B6 129SVJ mice^15,45^, whereas the level of AMPK phosphorylation was elevated in *Flcn*-deficient mouse embryonic fibroblasts and adipose-specific *Flcn* knockout mice^46,47^. Results from these earlier studies suggested that the manner in which *FLCN* regulates AMPK is context dependent. In the lungs of mice in which *Flcn* expression was conditionally deleted in alveolar epithelial cells, decreased membrane localization of E-cadherin and LKB1 that impaired AMPK activation was reported^15^. The consequences from this *Flcn* loss was increased alveolar epithelial apoptosis, which could be rescued by the AMPK activator AICAR or constitutively active AMPK.

Our results obtained in primary cultured BHDS-PMCs, although they suffered from *FLCN* haploinsufficiency but not complete *FLCN* loss, showed similar patterns to those reported in Goncharova et al.^15^. We demonstrated that AICAR reversed diminished AMPK phosphorylation and ameliorated apoptosis for 24 h, but not proliferation over a 3-day period, in BHDS-PMCs. Our findings imply that the restoration of AMPK phosphorylation is not enough to improve long-term cell survival in BHDS-PMCs. We speculate that this is partially because folliculin has so many binding partners, and may select one or more unique partner(s) depending on the differentiation stage and nutrient signaling^48,49^.

Furthermore, a long-lasting state of *FLCN* haploinsufficiency, rather than abrupt null *FLCN* expression by deletion, may cause different intracellular signaling pathways to keep cellular homeostasis. This may occur due to *FLCN* regulating mTOR complex 1/2 and Wnt signaling pathways in early human pluripotency, as was recently reported^48^. In this context, the amount of normal folliculin protein required for maintaining cellular homeostasis could change depending on cell type, cell differentiation stage, nutritional environment, etc. Further experiments are needed to investigate cells constituting target organs of BHDS (i.e., skin follicles, lungs, and kidneys), and how the *FLCN* haploinsufficiency state affects cellular function.

This study had several limitations. Our data consist only of those generated from human primary cultured cells. It is typically difficult to maintain BHDS-PMCs for extended periods. BHDS-PMCs show reproducibly poor viability at the second passage, so all assays were performed using BHDS-PMCs at the first passage after FACS. We performed gene expression microarray analysis using the *FLCN*-knockdown human pleural MC line MeT-5A (American Type Culture Collection, Manassas, VA, USA), in which *FLCN* was depleted by using small hairpin RNA (shRNA). However, shRNA-treated MeT-5A showed a fourfold upregulation of the *CDH1* expression level compared with that in control MeT-5A (data not shown). The methods to immortalize PMCs with various physiological characteristics retained, if available, would greatly facilitate the research in this field.

In addition, we used PSP-PMCs as a control from the viewpoint that they were not BHDS-PMCs and expected to carry wild-type *FLCN*. However, we had no way to obtain PMCs from individuals without lung diseases. Our eligibility criteria for participants with PSP were relatively young patients without any underlying lung diseases except for lung bullae on the apical segments of the upper and lower lobes. Since bullae are generally focal, we believe that most of the PMCs we obtained using FACS would be normal and therefore were acceptable to utilize as a control. We also focused only on the downregulation of E-cadherin expression caused by *FLCN* haploinsufficiency from the results of gene expression analyses. PMCs play a critical role in normal serosal repair following injury^50^, therefore, impaired wound healing might lead to disruption of the mesothelium in addition to the fragility of cell–cell junctions. Further investigation is warranted to explore the development of pneumothorax from the perspective of tissue repair response in a state of *FLCN* haploinsufficiency.

In conclusion, we found that *FLCN* haploinsufficiency impaired fundamental cellular functions of PMCs, in which abnormalities of the E-cadherin-LKB1-AMPK signaling pathway were involved at a minimum. We believe this study is a steppingstone to elucidate the mechanisms by which disruption of the mesothelium causes pneumothorax as well as to identify treatable targets to prevent mesothelium disruption.

## Supplementary information

Supplementary Informations.
