# Supplementary material for: Folliculin haploinsufficiency causes cellular dysfunction of pleural mesothelial cells
Source: Sci Rep. 2021 May 24;11:10814. doi: 10.1038/s41598-021-90184-9 (PMC8144428; doi:10.1038/s41598-021-90184-9)
Supplement: Supplementary file 1 — Supplementary Informations. [file 41598_2021_90184_MOESM1_ESM.docx]

**Supplementary Information**

**Folliculin Haploinsufficiency Causes Cellular Dysfunction of Pleural Mesothelial Cells**

**Author names and affiliations:**

Shouichi Okamoto^1,3,*^, Hiroki Ebana^3,4^, Masatoshi Kurihara^3,5^, Keiko Mitani^1,3^, Etsuko Kobayashi^1,3^, Takuo Hayashi^2,3^, Yasuhito Sekimoto^1,3^, Koichi Nishino^1,3^, Mizuto Otsuji^3,4^, Toshio Kumasaka^3,6^, Kazuhisa Takahashi^1^, Kuniaki Seyama^1,3^

^1^Division of Respiratory Medicine, ^2^Department of Human Pathology, Juntendo University Faculty of Medicine and Graduate School of Medicine, Tokyo, 113-8431, Japan

^3^The Study Group for Pneumothorax and Cystic Lung Diseases, Tokyo, 158-0095, Japan

^4^Department of Thoracic Surgery, Tokyo Metropolitan Bokutoh Hospital, Tokyo, 130-8575, Japan

^5^Pneumothorax Research Center and Department of General Thoracic Surgery, Tamagawa Hospital, Nissan Institute of Medicine, Tokyo, 158-0095, Japan

^6^Department of Pathology, Japanese Red Cross Medical Center, Tokyo, 150-8935, Japan

**Corresponding author:**

Shouichi Okamoto, E-mail: [sho-okamoto@juntendo.ac.jp](mailto:sho-okamoto@juntendo.ac.jp)

**Supplemental information for Materials and Methods**

Reverse transcription polymerase chain reaction (RT-PCR) and quantitative real-time reverse transcription PCR (qRT-PCR)

Total RNA was isolated using a RNeasy Plus Mini Kit (Qiagen, Hilden, Germany) according to the manufacturer’s protocol. Complementary DNA (cDNA) was created from total RNA (100 μg) using a SuperScript VILO cDNA Synthesis Kit (Invitrogen, Carlsbad, CA, USA). Polymerase chain reaction (PCR) was performed with AmpliTaq Gold DNA Polymerase (Thermo Fisher Scientific, Waltham, MA, USA) according to the manufacturer’s protocol. The following program was run for RT-PCR: holding at 94°C for 3 minutes and amplification by 35 cycles (denaturation at 94°C for 30 seconds, annealing at 55°C for 30 seconds, and extension at 72°C for 1 minute). For visualization of RT-PCR products, 12.5 μl of aliquot from a PCR reaction mixture and 2.5 μl of 6×Loading Dye (Toyobo Co., Ltd., Osaka, Japan) were fractionated in 2% agarose gel electrophoresis. The gel was photographed using a charge-coupled device camera, AE-6905 CF (ATTO Corporation, Tokyo, Japan).

qRT-PCR was conducted with Fast SYBR Green Master Mix (Applied Biosystems, Carlsbad, CA, USA) according to the manufacturer’s instructions. The PCR program for qRT-PCR was completed as follows: holding at 95°C for 20 seconds, amplification by 40 cycles (denaturation at 95°C for 3 seconds, annealing and extension at 60°C for 30 seconds), and a melt-curve analysis. All reactions were run in triplicate using the *GAPDH* (also known as Glyceraldehyde-3-Phosphate Dehydrogenase) gene as an internal standard.

Detachment assay

Pleural mesothelial cells (PMCs) were seeded onto a collagen-coated 12-well plate (Corning Incorporated, Corning, NY, USA) at a density of 1×10^5^ cells/well (in 3 replicate wells). Cells with 90% confluence were gently washed with calcium-free phosphate-buffered saline twice and incubated with 1 mM ethyleneglycol-*bis*-(β-aminoethyl ether)-*N,N,N’,N’*-tetraacetic acid (pH 8.0) for 20 minutes. Supernatant was collected and the cell number was measured after centrifuge at 1,500 rpm for 5 minutes. Residual cells in the wells were dissociated using 0.05% trypsin/0.2 mM ethylenediaminetetraacetic acid (EDTA) for 5 minutes and these cells were also counted. The percentage of detached cells was computed by dividing the number of detached cells by the total number of detached plus residual cells. An average percentage of the 3 replicates in each sample (PSP, n = 6; BHDS, n = 6) was recorded as the representative value.

Apoptosis assay

Cell apoptosis was evaluated with flow cytometry using a fluorescein isothiocyanate (FITC)-conjugated Annexin V Apoptosis Detection Kit I (BD Biosciences, San Jose, CA, USA) according to the manufacturer’s instructions. PMCs were seeded onto a collagen-coated 12-well plate (Corning Incorporated) at a density of 1×10^5^ cells/well (in 3 replicate wells). Cultured PMCs with 90% confluence were incubated in complete medium with or without 0.5 mM 5-aminoimidazole-4-carboxamide ribonucleotide ([AICAR]; Wako Pure Chemical Ind. Ltd., Osaka, Japan) for 24 hours, then were dissociated using 0.05% trypsin/0.2 mM EDTA for 5 minutes. Cells positive for both FITC-conjugated annexin V and propidium iodide were regarded as apoptotic cells. Fluorescent intensity was analyzed using a BD LSRFortessa cell analyzer (BD Biosciences). The effect of incubating PMCs with AICAR for 24 hours on PMCs apoptosis was also evaluated. The mean percentage of the 3 replicates in each sample (PSP, n = 5; BHDS, n = 4) was recorded as the representative value.

Western blot analysis of E-cadherin-liver kinaseB1(LKB1)-5’AMP-activated protein kinase (AMPK) signaling pathway

PMCs were seeded at a density of 3×10^5^ cells on a collagen-coated 6-well plate (Corning Incorporated). After cells reached 90% confluence, the complete medium was changed into serum-free medium, and cells were incubated for 24 hours in the absence or presence of 1 mM AICAR. Then, cells were lysed at the indicated times with radioimmunoprecipitation assay buffer (Thermo Fisher Scientific) supplemented with protease and phosphatase inhibitors (Thermo Fisher Scientific). Protein concentration was determined using a Pierce BCA Protein Assay Kit (Thermo Fisher Scientific). Sodium dodecyl sulfate-polyacrylamide gel electrophoresis was performed using a 4-20% gradient polyacrylamide gel (Bio-Rad, Hercules, CA, USA), and separated proteins were transferred to a PVDF membrane (Merck Millipore, Bedford, MA). The membrane was probed with rabbit anti-AMPK, anti-phospho AMPKα (Thr172), anti-LKB1, anti-phospho-LKB1 (Ser428), anti-β-actin, or mouse anti-E-cadherin primary antibodies. After overnight incubation at 4°C, the membranes were incubated again with relevant diluted peroxidase-conjugated secondary antibodies. Signal was detected using the Pierce ECL Western Blotting Substrate (Thermo Fisher Scientific), and the band intensities were quantified using the Fiji program of ImageJ software.

**Supplementary Table S1. *FLCN* mutations of study participants with BHDS and usage of isolated PMCs**

| **Age** | **Sex** | **Location** | **Mutation** | **Usage of Samples*** |
| --- | --- | --- | --- | --- |
| 45 | Female | Exon 12 | c.1347_1353dupCCACCCT | (1), (9) |
| 36 | Female | Exon 11 | c.1285dupC | (1), (5) |
| 23 | Female | Exon 5 | c.318 C>G | (1) |
| 34 | Male | Exon 7 | c.769_771delTCC | (2), (3), (7) |
| 44 | Female | Exon 12 | c.1347_1353dupCCACCCT | (2), (4), (5), (7), (9) |
| 39 | Female | Intron 11 | c.1301-2A>G | (2), (12) |
| 32 | Male | Exon 12 | c.1347_1353dupCCACCCT | (3), (5), (8), (12) |
| 46 | Female | Exon 11 | c.1285dupC | (3), (6), (8), (10), (11), (12) |
| 48 | Female | Exon 12 | c.1429C>T | (4), (5), (7) |
| 44 | Female | Exon 12 | c.1347_1353dupCCACCCT | (4), (5), (6), (8), (10), (11), (12) |
| 29 | Male | Exon 13 | c.1533_1536delGATG | (5), (6), (9), (10), (11), (12) |
| 44 | Male | Exon 1** | c.1-455-?_c.1-228+?del | (6), (8), (12) |

*The descriptions of usage of samples for experiments are as follows: (1), microarray analysis and gene set enrichment analysis; (2), scanning electron microscopy; (3), transmission electron microscopy; (4), flow cytometric analysis of E-cadherin; (5), detachment assay; (6), cell proliferation assay; (7), wound cell migration assay; (8), apoptosis assay by flowcytometry; (9), Immunohistochemical staining of cleaved caspase-3; (10), Phalloidin immunofluorescence staining; (11), measurement of guanosine triphosphate (GTP)-bound Ras homolog family member A (RhoA) and RhoA activity; and (12), western blot analysis.

**The description of the exon 1 mutation indicates a large genomic deletion including the entire exon, but the exact breakpoints were not determined.

**Supplementary Table S2. List of antibodies used for flow cytometric analysis**

| **Antibody** | **Conjugate** | **Clone** | **Catalog No.** | **Supplier** |
| --- | --- | --- | --- | --- |
| Anti-E-cadherin | Allophycocyanin | 67A4 | 324107 | BioLegend |
| Anti-mesothelin |  | 22A31 | 10357 | Immuno-Biological Laboratories |
| Anti-podoplanin | Alexa Fluor 647 | NC-08 | 337008 | BioLegend |
| Mouse IgG1 κ | Biotin | P3.6.2.8.1 | 13-4714-85 | Thermo Fisher Scientific |
| Mouse IgG1 κ | Allophycocyanin | MOPC-21 | 400121 | BioLegend |
| Rat IgG2a κ | Alexa Fluor 647 | RTK-2758 | 400526 | BioLegend |

**Supplementary Table S3. List of primers used for RT-PCR analysis**

| **Gene** | **Transcript** | **Primer Sequence (5’-3’)** |
| --- | --- | --- |
| *CALB2* | Calretinin | F: ACATTGACGAGCATGAGCTG  R: CCAAGGACATGACGCTCTTT |
| *GAPDH* | GAPDH | F: GAGCCTGAGCTGTGAGGTCT  R: GAAGGTGTGGCTGAACACAA |
| *KRT5* | Keratin 5 | F: GGAGCTCATGAACACCAAGC  R: TGCCATATCCAGAGGAAACA |
| *VIM* | Vimentin | F: TCTGGATTCACTCCCTCTGG-  R: GGTCATCGTGATGCTGAGA |
| *WT1* | Wilms’ tumor 1 | F: AAGACCCACACCAGGACTCA  R: TGTGATGGCGGACTAATTCA |

Abbreviations: F = forward primer; R = reverse primer.

**Supplementary Table S4. List of primers used for qRT-PCR analysis**

| **Gene** | **Transcript** | **Primer Sequence (5’-3’)** |
| --- | --- | --- |
| *CDH1* | E-cadherin | F: GACTCGTAACGACGTTGCAC  R: GGTCAGTATCAGCCGCTTTC |
| *DSG2* | Desmoglein-2 | F: CTAACAGGTTACGCTTTGGATGC  R: GTGAACACTGGTTCGTTGTCAT |
| *FLCN* | Folliculin | F: GAGCCTGAGCTGTGAGGTCT  R: GAAGGTGTGGCTGAACACAA |
| *GAPDH* | GAPDH | F: GGTCTCCTCTGACTTCAACA  R: GTGAGGGTCTCTCTTCCT |
| *OCLN* | Occludin | F: TGGCTGCTGCTGATGAATAC  R: ACAACTTGGCATCAGCCTTC |
| *PKP4* | p0071 | F: CATCATTCAGTCAGGCTCCA  R: GGAAAGTGAACTCGGTCATCA |
| *TJP1* | ZO-1 | F: TTACCACACTGTGCGTCCAT  R: CACAGTTTGCTCCAACGAGA |

Abbreviations: F = forward primer; R = reverse primer; ZO-1 =

zonula occludens-1.

**Supplementary Table S5. List of antibodies used for Western blot analysis**

| **Antibody** | **Catalog No.** | **Supplier** |
| --- | --- | --- |
| Anti-AMPK | 2532 | Cell Signaling Technology |
| Anti-Phospho-AMPK α (Thr172) | 2535 | Cell Signaling Technology |
| Anti-E-cadherin | 610181 | BD Biosciences |
| Anti-LKB1 | 3050 | Cell Signaling Technology |
| Anti-Phospho-LKB1 (Ser428) | 3482 | Cell Signaling Technology |
| Anti-β-actin | 4970 | Cell Signaling Technology |

All antibodies were used at 1:1000 dilution.

**Supplementary Table S6. Representative Gene Ontology (GO) terms identified to be statistically significant by gene expression microarray analysis**

| **GO Accession No**. | **GO Term** | ***p*** | ***Q*** |
| --- | --- | --- | --- |
| GO Terms including “Adhesion” | | | |
| GO: 0022610 | Biological Adhesion | 1.69x10^-12^ | 4.64x10^-9^ |
| GO: 0007155 | Cell Adhesion | 4.35x10^-12^ | 1.13x10^-8^ |
| GO: 0030155 | Regulation of Cell Adhesion | 2.22x10^-8^ | 2.23x10^-5^ |
| GO: 0045785 | Positive Regulation of Cell Adhesion | 2.23x10^-7^ | 1.77x10^-4^ |
| GO Terms including “Cell Proliferation” | | | |
| GO: 0042127 | Regulation of Cell Proliferation | 4.69x10^-8^ | 4.39x10^-5^ |
| GO: 0008285 | Negative Regulation of Cell Proliferation | 1.39x10^-7^ | 1.15x10^-4^ |
| GO: 0050678 | Regulation of Epithelial Cell Proliferation | 2.93x10^-7^ | 2.26x10^-4^ |
| GO Terms including “Cell Migration” | | | |
| GO: 0016477 | Cell Migration | 8.68x10^-13^ | 2.62x10^-9^ |
| GO: 0030334 | Regulation of Cell Migration | 1.54x10^-14^ | 7.19x10^-11^ |

GO terms that were statistically significant (*p*<0.01 and FDR, *Q*<0.001) were selected from 3,378 genes with a fold change >1.5, using GeneSpring14.9.1. Abbreviations: FDR = False Discovery Rate; GO = Gene Ontology.

**Supplementary Fig S1. Dendrogram of 3 PSP- and BHDS-PMCs samples**

0

Color range


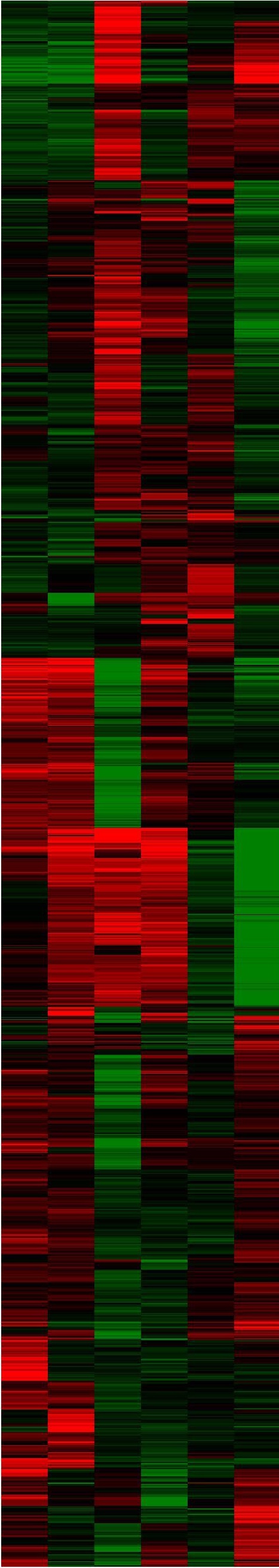

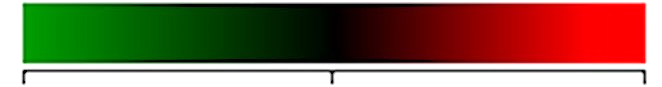


-2

2

BHDS1

BHDS2

BHDS3

PSP3

PSP2

PSP1

Hierarchical clustering analysis in PSP-PMCs (n = 3) and BHDS-PMCs samples (n = 3). A dendrogram, consisting of 1,799 genes with a fold change >1.5, shows the grouping of genes based on the similarity between them. Increased and decreased gene expression is shown from red to green, respectively. The color-range bar indicates a log2 fold change. The analysis illustrated that 1 PSP-PMCs sample was clustered into BHDS-PMCs group. The data were processed and analyzed using GeneSpring14.9.1 (http://genespring-support.com/).

**Full RT-PCR image of Figure 2C**

**
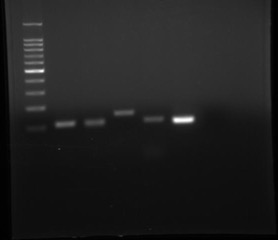
**

500 bp −

WT1

Calretinin

Keratin 5

Vimentin

GAPDH

**Full blot image of Figure 6A**

PSP

BHDS

BHDS

PSP

pAMPK

62 kDa −

Serum starvation, AICAR (-)

AMPK

62 kDa −

**
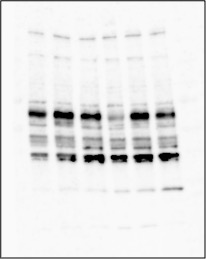
**

**
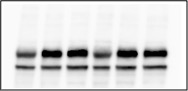
**

**Full blot image of Figure 6A**

Serum starvation, AICAR (-)

**
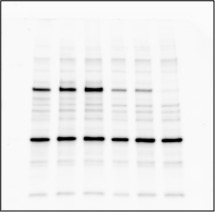
**

E-cadherin

PSP

BHDS

120 kDa −

**
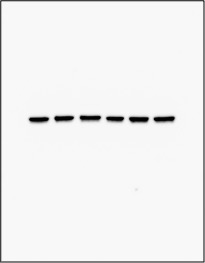
**

PSP

BHDS

β-actin

45 kDa −

**Full blot image of Figure 6A**

Serum starvation, AICAR (+)

**
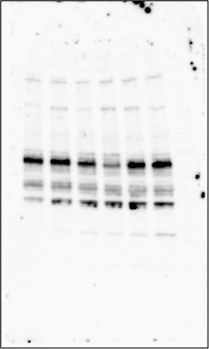
**

PSP

BHDS

62 kDa −

pAMPK

**Full blot image of Figure 6A**

Serum starvation, AICAR (+)

**
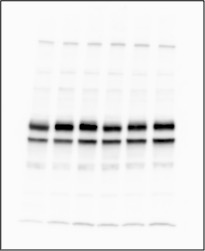
**

PSP

BHDS

62 kDa −

AMPK

**Full blot image of Figure 6A**

Serum starvation, AICAR (+)

BHDS

PSP

**
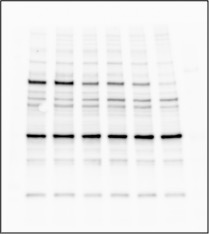
**

E-cadherin

120 kDa −

**
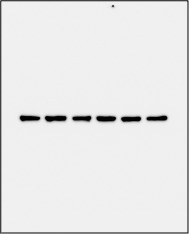
**

BHDS

PSP

45 kDa −

β-actin

**Full blot image of Figure 6D**

**
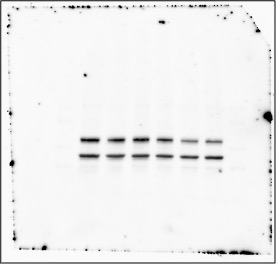
**

PSP

BHDS

54 kDa −

pLKB1

**
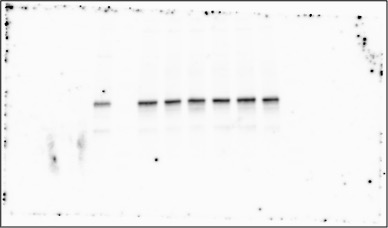
**

BHDS

PSP

PC-9

LKB1

54 kDa −

**Full blot image of Figure 6D**

**
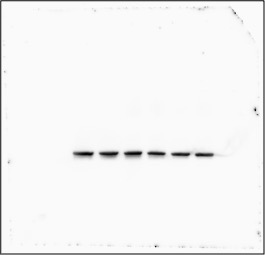
**

BHDS

PSP

β-actin

45 kDa −
